# Group 1 LEA Proteins in Durum Wheat: Evolution, Expression, and Roles in Abiotic Stress Tolerance

**DOI:** 10.3390/plants14182817

**Published:** 2025-09-09

**Authors:** Najeh Soltani, Ikram Zaidi, Mohamed Najib Saidi, Faiçal Brini

**Affiliations:** Biotechnology and Plant Improvement Laboratory, Centre of Biotechnology of Sfax, P.O. Box 1177, Sfax 3018, Tunisia; najeh.soltani@cbs.rnrt.tn (N.S.); mohamednajib.saidi@cbs.rnrt.tn (M.N.S.)

**Keywords:** abiotic stress, durum wheat, *EM* genes, expression analysis, LDH, Yeast

## Abstract

Group 1 LEA proteins are involved in embryo water dynamics during the maturation stage of seed development and contribute to desiccation stress protection in vegetative and embryonic tissues. Nevertheless, their roles in durum wheat remain largely unexplored. This study represents the first comprehensive survey of group 1 LEA proteins and their encoding genes in *Triticum turgidum* ssp. Durum (durum wheat). Eight group 1 *LEA* (*TtEM1* to *TtEM8)* genes were identified in the durum wheat genome, which were named according to their chromosomal location. Analyses of the physiochemical characteristics and subcellular location revealed that all *TtEM* proteins exhibited a highly disordered structure (more than 90% of tendency of disorder) and were located in the nucleus. Evolutionary analysis between the durum wheat family and all other known group 1 LEA proteins from *Arabidopsis thaliana*, rice (*Oryza sativa*), barley (*Hordeum vulgare*), and barrel medic (*Medicago truncatula*) showed four phylogenetic groups; each group shares the same conserved motifs and gene structure. Interestingly, almost *TtEM* genes harbor cis-elements related to hormone regulation, stress response, and growth regulation, indicating their function in stress tolerance and developmental control. Subsequently, Expression analysis of two homoeologous genes, *TtEM1* and *TtEM4*, demonstrated that the two genes exhibited distinct expression profiles across different tissues and in response to various stress treatments, suggesting that these genes may be involved in regulating growth, development, and stress adaptation in durum wheat. *TtEM1* and *TtEM4* purified proteins act as molecular chaperones and protect LDH activity against desiccation, cold, and heat treatments. Moreover, *TtEM1* and *TtEM4* genes were proved to enhance heat, cold, oxidative, and drought tolerance in yeast. These results clearly described the characteristics and the evolutionary dynamics of the *EM* gene family in wheat, and unveiled their role in wheat development and response to abiotic stress.

## 1. Introduction

Durum wheat (*Triticum turgidum* ssp. durum) is a major food crop, especially in the Mediterranean region, where it is largely produced and exploited [1]. Nevertheless, as sessile organism, Durum wheat is frequently exposed to different stress conditions such as high temperatures, drought, salinity, and cold during its development and its growth, thus impacting global wheat production. To cope with these adverse conditions, plants have developed a variety of molecular and physiological defense mechanisms to survive in hostile environments [2]. LEA (Late Embryogenesis Abundant) proteins are among the crucial stress-responsive proteins [3]. LEA proteins were first identified during the late stages of embryonic development in cotton seeds [4]. They constitute a multifaceted family of small (10–30 kDa) hydrophilic and intrinsically disordered proteins, and they undergo a disorder-to-order structure when interacting with specific molecules or under stress conditions that is considered to be essential for their protective role [5]. This group of proteins was recognized to be involved in abiotic stress tolerance. As demonstrated, LEA proteins play a crucial role in stabilizing membranes by interacting with phospholipids under stress, preserving their structure and maintaining physical membrane integrity [6]. Furthermore, these proteins have been regarded as preventing the inactivation of different enzymes, such as lactate dehydrogenase, malate dehydrogenase, citrate synthase, or catalase, under stress conditions [7]. In addition, some LEA proteins scavenge reactive oxygen species and bind different metal ions, thereby reducing ion toxicity and oxidative damage under stress [8]. Recently, LEA proteins from different organisms have been observed to undergo LLPS (liquid-liquid phase separation) to promote water stress tolerance [9]. According to their conserved motifs, LEA proteins are classified into eight families in the PFAM database: dehydrin, LEA_1, LEA_2, LEA_3, LEA_4, LEA_5, LEA_6, and seed maturation proteins (SMP), corresponding to eight PFAMs (PF00257, PF03760, PF03168, PF03242, PF02987, PF00477, PF10714, and PF04927) [10]. The LEA_5 family members are clearly distinct from other LEA proteins. They are found in organisms from all three domains of life, namely eukarya, archaea, and bacteria, and they are very hydrophilic and highly conserved through the whole length of the proteins [11]. They are also considered with the LEA_2 and LEA_4 families as true LEA proteins, including most members of the protein family [12].

Group1 (LEA_5, Dur-19, Em (Early methionine), PF00477) LEA proteins, were initially identified as major embryonic proteins when poly A RNA was isolated from a dry wheat embryo and translated in vitro in the presence of 35S-methionine [13]. Over 100 *Em* genes have been characterized in different species, including plants, bacteria, and brine shrimp [14]. In plants, *Em* genes have been identified in different plant species like *Arabidopsis*, rice, maize, soybean, black locust, lettuce, papaya, castor bean, cassava, and tigernut [15]. It has been demonstrated that Em proteins contain a conserved 20-amino acid motif (TRKEQ [L/M]G[T/E]EGY[Q/K]EMGRKGG[L/E]) present as tandem repeats from one to four copies in plant species and from one to seven copies in other organisms, Em proteins also present two other conserved regions, an N-terminal sequence and C-terminal sequence [16]. The observation of Em proteins having a high proportion of Gly, Glu, and Gln amino acids, and the absence of tryptophan and cysteine residues offer insight into their disordered structure and their role to mitigate stress damage caused by freezing, desiccation, and osmotic stress [11]. Expression analyses have shown that *Em* genes are strongly regulated and are mainly induced in mature embryos, as well as in vegetative tissues in response to abscisic acid (ABA) and osmotic stress [17]. In lettuce, the *LsEm1* gene was accumulated during the flowering and mature stage, and also in response to cold, salt, dehydration, and ABA in young seedlings [18]. *OsEm1*gene has been shown to be responsive to different abiotic stresses, such as drought, salinity, cold, and ABA treatment, at vegetative stages in rice [14]. *CeLEA5* genes from tigernut exhibit predominant expression in tubers and display a seed desiccation-like accumulation throughout tuber maturation [19]. Evidence from different studies revealed that overexpression of *Em* genes can enhance tolerance to abiotic stresses. For instance, the expression of *OsEm1* in rice enhances tolerance to drought stress [14]. Likewise, overexpression of the *Arabidopsis AtEm6* gene improved salt tolerance in transgenic rice cell lines [20]. Additionally, overexpressing *LsEm1* in rice enhances drought and salt tolerance by improving stress-related physiological traits and upregulating key genes like *OsCDPKs* and *rab21*, reducing yield loss under stress [18]. Given the critical role of *Em* genes in plants’ abiotic stress resistance, it is necessary to study *Em* in major crops like wheat. In this study, we identified and characterized eight group 1 LEA genes (*TtEM*) in durum wheat, distributed across four chromosomes. Using prediction tools, we analyzed their phylogenetic relationships, physicochemical properties, gene structures, conserved motifs, and upstream cis-regulatory elements. Furthermore, we investigated the expression profiles of two representative genes, *TtEM1* and *TtEM4*, across different tissues and under various stress conditions. Their potential function was further evaluated through assays measuring the protection of lactate dehydrogenase (LDH) activity and the enhanced stress tolerance of transformed yeast cells under osmotic, oxidative, and thermal stress conditions. The genome-wide characterization presented here provides a critical resource for engineering resistant wheat cultivars.

## 2. Results

### 2.1. Identification and Characterization of TtEM (LEA_5) Genes in Durum Wheat

Based on their IDs from the Pfam protein family database Pfam (PF00477), we identified eight genes encoding group 1 LEA proteins in durum wheat (*Triticum turgidum* ssp. Durum) and named according to their chromosomal locations (Table S1). The *Tt*EM proteins share three conserved motifs, as shown by protein sequence alignment using ClustalW (Figure S1). A first motif corresponding to the N-terminal part, a second motif corresponding to the C-terminal part, and a third motif of twenty amino acids specific for EM proteins (representing the PF00477 domain) repeated two to three times in tendency. Analysis of the physicochemical properties showed that the LEA proteins studied from group 1 in durum wheat share the same characteristics as LEA proteins (Table 1). In fact, the number of amino acids varies between 93 and 133 aa, and the molecular weight is between 9.9 and 14.6 KDa, indicating a low molecular weight, one of the characteristics of LEA proteins. According to the predicted pI values, which vary between 5.17 and 5.57, these proteins are acidic. The negative grand average of the hydropathy index suggested that TtEM proteins were hydrophilic. LEA proteins are predominantly disordered in the native state and are capable of acquiring secondary structures in the form of an alpha helix, which is shown by the SPOMA prediction tool, from 33 to 45% of predicted α-helical content, and the results obtained by the PONDR prediction tool show that approximately 98–100% of the residues that form these proteins are disordered. Analysis of their subcellular localization shows that all *Tt*EM proteins can be localized in the nucleus, which provides some indications of the function of this group of proteins.

### 2.2. Chromosome Distribution and Phylogenetic Analysis

**Figure 1:**
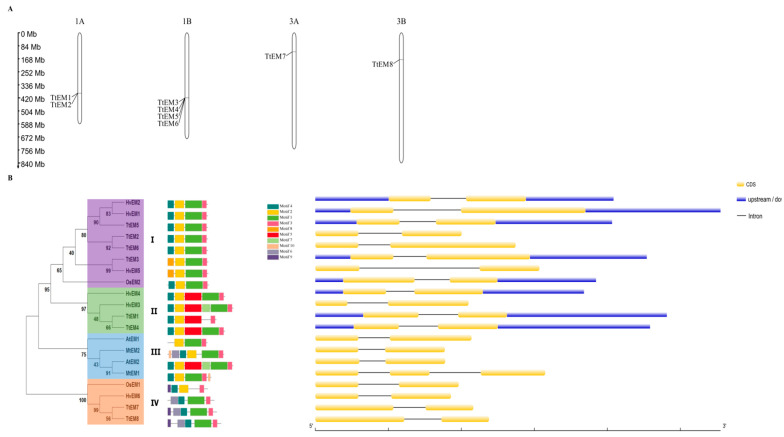
Chromosomal distribution and phylogenetic relationships. (**A**) Chromosome distributions of *TtEM* genes in *Triticum turgidum*. The scale of the chromosome is in millions of bases (Mb). (**B**) phylogenetic tree, conserved motifs, and gene structures of *TtEM* genes (*Triticum turgidum*), *HvEM* genes (*Hordeum vulgare*), *AtEM* genes (Arabidopsis *thaliana*), and *MtEM* genes (*Medicago truncatula*). The phylogenetic tree on the left side was constructed using MEGA.11. Four clades are represented in four different colors. Conserved motifs are counted and marked in colored boxes.

The eight *TtEM* genes are unevenly distributed across four chromosomes: two on chromosome 1A, four on chromosome 1B, only one gene on chromosome 3A, and only one on chromosome 3B (Figure 1A). In order to investigate their evolutionary relationships, a phylogenetic tree was generated with their homologous from barley (*Hordeum vulgare*), rice (*Oryza sativa*), *Medicago truncatula*, and *Arabidopsis thaliana* (Figure 1B). Their phylogenetic analysis revealed four distinctive groups, with durum wheat genes distributed across them, indicating evolutionary diversity. The fourth clade showed strong phylogenetic between barley and wheat genes, suggesting a common evolutionary history or similar selective pressures, as supported by the bootstrap values. The third clade consisted exclusively of *Arabidopsis thaliana* and *Medicago truncatula* genes, indicating greater divergence of the dicotyledons compared with the monocotyledons (represented by durum wheat and barley). The second clade contained only wheat and barley genes, consistent with their classification to the Triticeae tribe. Finally, the first clade demonstrated closer relationships with rice genes, suggesting another evolutionary pathway or conserved functions specific to these genes in durum wheat.

### 2.3. Gene Structure and Conserved Motif Analyses of EM Genes

In order to gain a better picture about the relationships of the durum wheat *EM* genes with the other *EM* genes, the exon–intron structure of *TtEM* genes was analyzed (Figure 1B). All of the *EM* genes contained one intron, except for one *EM* gene (*HvEM3* gene), which contained no intron. Additionally, the *HvEM3* gene contained one exon, while the remainder of the *EM* genes had two exons. Generally, the exon–intron structures of *EM* genes within the same group were similar, supporting their classification and phylogenetic relationships. A total of 10 conserved motifs were obtained from the MEME database (Table 2). Members of the same group had same composition and arrangement of motifs, and some motifs were detected in only one group (Figure 1B). For example, motif 9 and motif 6 were only identified in the fourth group of *EM* genes. All members contained motif 3. In addition, motif 1, motif 2, and motif 3 were also found in most members. Group 1 displays a highly conserved structure composed of three motifs: motif 2, motif 1, and motif 3. Notably, motif 1 and motif 3 are almost present in all the protein sequences analyzed, suggesting a degree of conservation either within group 1 of LEA proteins or within the LEA protein family. Motif 2 is also found in most of the protein sequences. The similarity in motif composition within each group implies potential function similarity among the proteins in the same group.

### 2.4. Analysis of Cis-Acting Elements in Promoters of TtEM Genes

**Figure 2:**
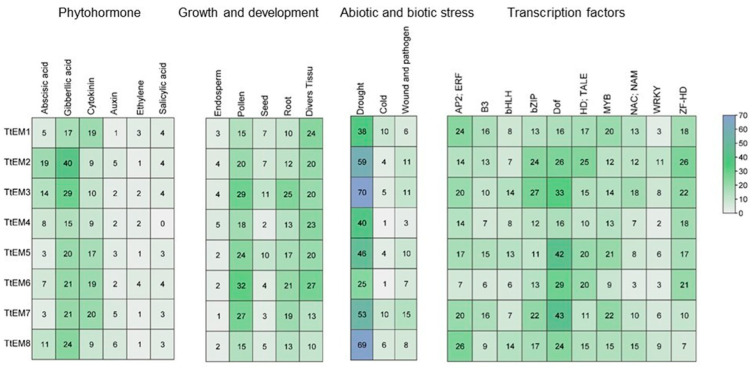
Predicted hormone response, abiotic and biotic stress response, transcription factors biding site, and growth- and development-related cis-elements within the promoter regions of *TtEM* genes. The values in the box represent the numbers of cis-elements.

To explore the potential functions of *TtEM* genes, we extracted the 2 kb upstream promoter sequences of each *TtEM* gene and analyzed them using the PLACE database to identify and quantify the cis-regulatory elements (Figure 2). Additionally, we used the Plant Promoter Analysis Navigator (PlantPAN) to identify transcription factors binding sites within these promoter regions. The identified cis-regulatory elements were classified into three main categories. The first category includes elements related to the hormone signaling pathway, mainly involved in plant growth and development processes, from seed germination to flowering, such as those responsive to gibberellin, abscisic acid, cytokinin, auxin, salicylic acid, and ethylene. The second category comprises stress-related cis-regulatory elements, particularly those associated with drought, low temperature, wounding, and pathogen responses. The third category includes elements related to plant growth and development, such as those involved in endosperm expression, pollen, and seed-specific regulation elements, indicating a strong relationship between the *TtEM* gene and seed expression patterns. Moreover, some cis-elements are linked to root- and tissue-specific expression (more details in Table S2). This classification indicates that *TtEM* genes can be involved in multiple biological processes, although some elements are more relevant than others. For instance, among phytohormone-responsive elements, those associated with gibberellic acid and cytokinin are the most widespread compared with the others. Similarly, within the development-related elements, those involved in pollen and root-specific expression are more frequent. Among the stress-responsive elements, drought-related elements are the most common, indicating a potential role for *TtEM* proteins in drought tolerance mechanisms in wheat. Furthermore, analysis of *TtEM* promoters revealed several transcription factors binding sites associated with plant development and stress responses. These include APETALA2/Ethylene Responsive Factor (AP2/ERF), B3, the Basic helix–loop–helix (bHLH), basic leucine zipper (bZIP), DNA binding with one finger (Dof), transcription activator-like effector (TALE), myeloblastosis (MYB), and Zinc finger homeodomain (ZF-HD) transcription factors. These regulators play crucial roles in signaling networks that modulate plant growth, development, and response to biotic and abiotic stresses.

### 2.5. Expression Patterns of TtEM1 and TtEM4 Genes in Different Tissues and Under Stress Treatments

**Figure 3:**
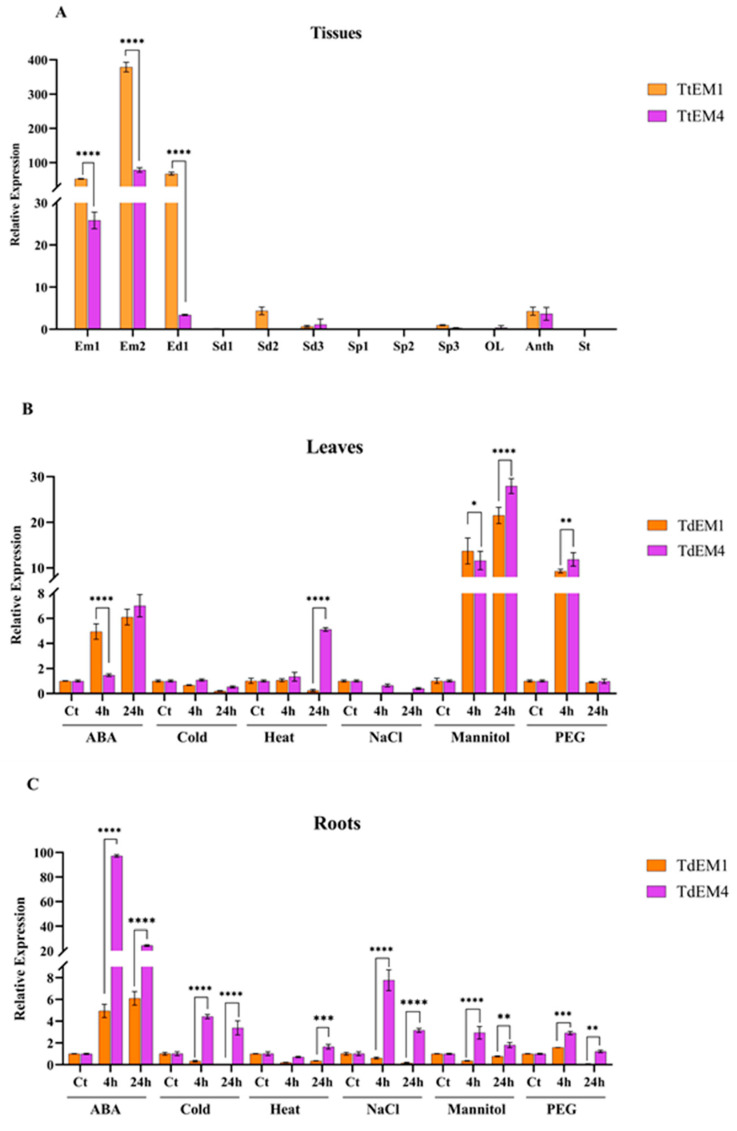
Relative expression levels of *TtEM1* and *TtEM4* genes. (**A**) Analysis of the expression patterns of *TtEM1* and *TtEM4* across different organs of Om Rabiaa variety after anthesis, ear (Sp1, after 2 days; Sp2, after 4 days; and Sp3, after 7 days), seeds (Sd1, after 4 days; Sd2, after 7 days; and Sd3, after 14 days), endosperm (Ed1, after 14 days), embryo (Em1, after 14 days; and Em2, after 21 days), leaves (OL), stems (St), and anthers (Ant). (**B**) Expression patterns of *TtEM1* and *TtEM4* in leaves of Om Rabiaa variety. (**C**) Expression profiles of *TtEM1* and *TtEM4* in roots of Om Rabiaa variety. (Ct) without treatment, after 4 h and 24 h of stress treatments with 100 μM ABA, cold (4 °C), heat (42 °C), 200 mM NaCl, 100 mM mannitol, and 20% PEG6000. Each bar value represents the mean ± SD based on triplicate experiments. Statistical significance of the difference was determined by two-way ANOVA: **** *p* < 0.0001, *** *p* < 0.001, ** *p* < 0.01, and * *p* < 0.05.

To elucidate the stress-responsive and organ-specific expression profiles of *TtEM* genes, we analyzed the expression profiles of two *TtEM* genes under abiotic stress conditions and across different tissues. In the embryo at 14 days after anthesis (Em1), *Tt*EM*1* and *TtEM4* genes were highly expressed; however, *TtEM1* showed a 2-fold higher expression level compared to *TtEM4*. At 21 days after anthesis (Em2), *Tt*EM*1* maintained a significantly higher expression (5-fold relative to *TtEM4*), suggesting that *TtEM1* is more actively transcribed in these tissues. In the endosperm (Ed1), the expression of *TtEM1* was approximately 20-fold higher than *TtEM4*. In the other tissues, both genes showed very low expression levels (<5-fold change). This was evident in seeds at two (Sd2) and three days (Sd3) after anthesis, as well as in anthers. In the remaining tissues, expression of both genes was either extremely low or absent, such as seeds at one day after anthesis (Sd1), in the spike one day (Sp1) and two days (Sp2) after anthesis, in leaves (OL), and in stems (St) (Figure 3A). These results suggest that *TtEM1* and *TtEM4* genes exhibit tissue-specific expression, which may be linked to their specific roles in development or in response to specific stimuli. *TtEM1* and *TtEM4* genes were generally upregulated in leaves in response to the different stress treatments, including ABA, heat, mannitol, and PEG, with varying expression levels depending on the stress type and exposure duration (4 h or 24 h). Under ABA treatment, *TtEM1* exhibited a higher induction, showing a 5-fold increase after 4 h, compared to a 1.4-fold increase for *TtEM4*. In contrast, under heat stress, the expression level of *TtEM4* (5-fold) was higher than that of *TtEM1* (0.2-fold). Mannitol treatment led to the upregulation of *TtEM1* and *TtEM4* genes at 4 h and 24 h of treatments, with the *TtEM4* gene displaying a significantly higher induction at 24 h (28-fold) compared to *TtEM1* (21-fold). Under PEG treatment, both genes were significantly induced at 4 h of treatment, but the *TtEM4* gene showed a slightly higher induction (12-fold) compared to *TtEM1* (9-fold). Under cold and salinity conditions, expression of *TtEM1* and *TtEM4* genes remained near control levels (≤1-fold; Figure 3B). In roots, *TtEM4* gene expression was consistently higher than that of *TtEM1*. Notably, under ABA treatment, *TtEM4* expression was strongly induced, showed a ~98-fold change at 4 h, and then decreased to 24-fold at 24 h, while *TtEM1* showed a 4-fold increase at 4 h, and 6-fold at 24. Similarly, under salt stress, *TtEM4* exhibited a notable induction (7.7-fold at 4 h and 3.1-fold at 24 h), whereas *TtEM1* showed negligible induction (0.6-fold at 4 h and nearly undetectable at 24 h). Although cold, heat, mannitol, and PEG treatments slightly induced the expression of *TtEM4* gene in roots, its transcript levels remained higher than the expression of *TtEM1* under the same conditions. For instance, under cold stress, *TtEM4* showed a 4.4-fold increase at 4 h and 3.3-fold at 24 h, whereas *TtEM1* exhibited minimal expression (0.3-fold at 4 h and only 0.02-fold at 24 h). Similarly, in response to mannitol, *TtEM4* was upregulated to 2.9-fold at 4 h and 1.8-fold at 24 h, while *TtEM1* displayed lower expression levels (0.3-fold and 0.7-fold, respectively; Figure 3C). Overall, *TtEM1* and *TtEM4* display different expression profiles in leaves and roots in response to different stresses. *TtEM4* appears to be more responsive to different abiotic stresses than *TtEM1*, showing higher expression levels, particularly after 4 h of treatment. These differences suggest that *TtEM1* and *TtEM4* may play distinct roles in the response of wheat to abiotic stresses.

### 2.6. Expression, Purification, and Structural Characterization of TtEM1 and TtEM4 Proteins

**Figure 4:**
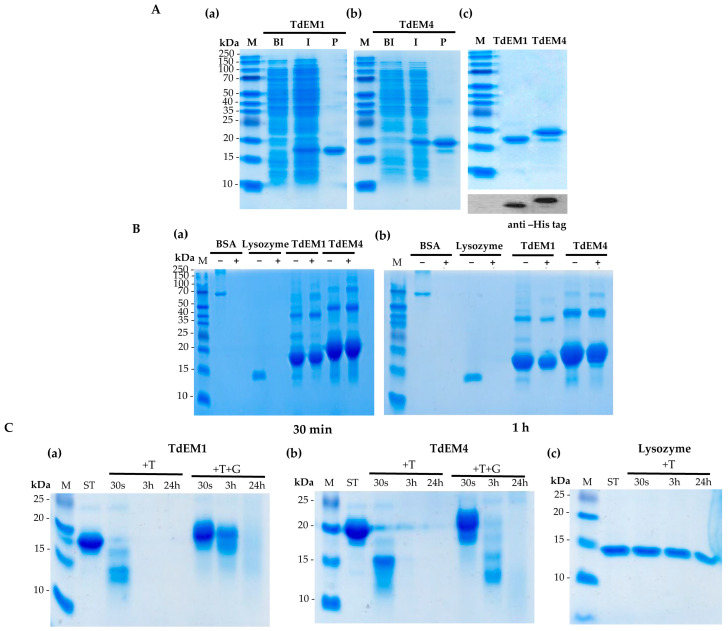
Analysis of the intrinsically disordered nature of *TtEM1* and *TtEM4* proteins. (**A**) SDS-PAGE analysis of *TtEM1* (**a**) and *TtEM4* (**b**) overexposed in *E*. coli BL21strain and Western blot analysis using a specific antibody anti-His (**c**). M, protein marker. BI, cells transformed with pET28a (+)-*TtEM1or TtEM4* before induction; AI, cells transformed with pET28a (+)-*TtEM1orTtEM4* after induction; P, purified *Tt*EM1 or *Tt*EM4 protein. (**B**) Heat stability of *TtEM1* and *TtEM4*: *TtEM1*, *Tt*EM4, BSA, and lysozyme were (+) or were not (−) incubated at 100 °C for 30 min (**a**) and for one hour (**b**). The supernatants were run on SDS-PAGE after centrifugation. (**C**) Limited proteolysis of *TtEM1* (**a**) or *TtEM4* (**b**) and lysozyme (**c**) with thermolysin (T). Incubation was performed for 30 s, 3 h, and 24 h, (−) without treatment, (T) incubated with thermolysin, and (+T+G) incubated with thermolysin and glycerol (25%). The reactions were stopped by adding SDS-PAGE loading buffer and heating for 5 min at 100 °C.

The *TtEM1* and *TtEM4* proteins were overexpressed in a bacterial host system to enable their expression and characterization. Following IPTG induction, two distinct bands corresponding to recombinant *TtEM1* and *TtEM4* were detected in the supernatant fraction (I) by SDS-PAGE that were absent before induction (BI; Figure 4A(a,b)). The proteins were subsequently purified, and their identities were confirmed by Western blot analysis using an anti-HIS antibody. The *TtEM1* protein migrated at an apparent molecular weight of 17 kDa, while its predicted size is 12.3 kDa. Similarly, *TtEM4* protein migrated at approximately 20 kDa, although its predicted size is 14.6 kDa; this abnormal migration is characteristic of intrinsically disordered proteins, which tend to exhibit abnormal SDS-PAGE behavior due to their low affinity for SDS (Figure 4A). The heat stability of the proteins was assessed by heating them at 100 °C. Both *TtEM1* and *TtEM2* remained in the supernatant after 30 min and even after 1 h of incubation, whereas the BSA and lysozyme used as structured proteins denatured and disappeared from the supernatant (Figure 4B(a,b)). Due to their lack of stable structure, intrinsically disordered proteins have more accessible and flexible regions, making them more susceptible to proteolytic cleavage. Consistent with this, both *TtEM1* and *TtEM4* exhibited sensitivity to thermolysin, as shown by SDS-PAGE analysis. Degradation began immediately upon the addition of thermolysin and was complete after 24 h, while lysozyme remained intact even after 24 h of treatment (Figure 4C(a–c)). To assess the impact of dehydration-mimicking conditions, 25% of glycerol was added. Under these conditions, *TtEM1* and *TtEM4* proteins showed increased resistance to thermolysin digestion. Although some degradation fragments were observed after 3 h of incubation, the proteins were largely protected, with complete degradation observed only after 24 h of treatment (Figure 4C(a,b)). These results suggest that glycerol-induced dehydration may promote structural transitions that enhance the protease resistance of *TtEM1* and *TtEM4*.

### 2.7. Preservation of LDH Activity Under Different Stress Treatments

**Figure 5:**
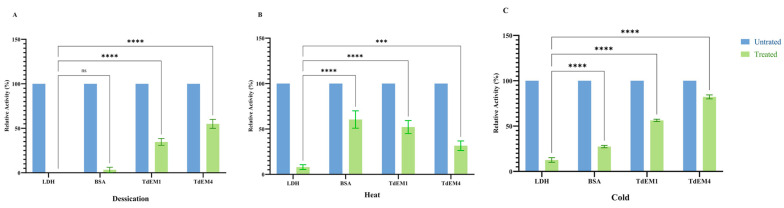
Protection of lactate dehydrogenase (LDH) by *TtEM1* and *TtEM4* proteins under stress treatment. (**A**) LDH activity measured after desiccation in presence and absence of *TtEM1* and *TtEM4* proteins. (**B**) After 30 min of heat treatment in 45 °C. (**C**) After two freeze–thaw cycles. BSA was used as positive control, and the enzymatic activity of non-treated LDH is referred to as 100%. Each column represents the mean of three replicates, and error bars indicate the standard deviation. Statistical significance of the difference was determined by two-way ANOVA: **** *p* < 0.0001, *** *p* < 0.001, and ns is *p* > 0.05.

We evaluated the ability of *TtEM1* and *TtEM4* to prevent damage to LDH activity under desiccation, heat, and freezing stress. BSA was used as a positive control. Under desiccation conditions, LDH completely lost its enzymatic activity. However, in the presence of *TtEM1* and *TtEM4*, approximately 34% and 55% of LDH activity was retained, respectively (Figure 5A). After incubation at 45 °C, LDH retained only 8% of its initial activity, but in the presence of *TtEM1* and *TtEM4*, 52% and 31% of its activity was retained, respectively (Figure 5B). In addition, we studied the effect of cold by freezing and thawing the samples. LDH activity decreased to around 12%, but with the addition of *TtEM1* and *TtEM4*, 56% and 82% of LDH activity was retained, respectively (Figure 5C). The results suggest that *TtEM1* and *TtEM4* proteins confer differential protective effects on LDH activity, particularly under cold and desiccation stress, and they are more effective than BSA under these conditions.

### 2.8. Overexpression of TtEM1 and TtEM4 Enhanced Yeast Cell Tolerance to Abiotic Stress

**Figure 6:**
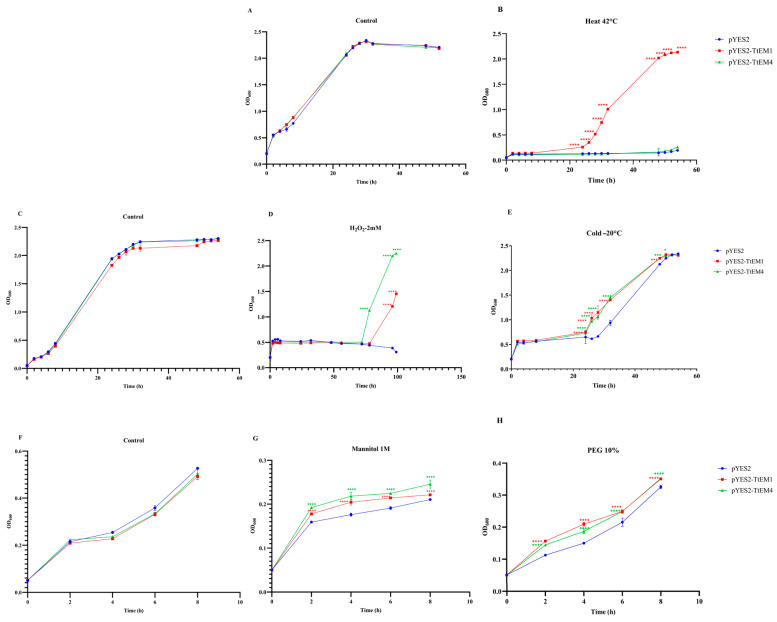
Overexpression enhances tolerance to abiotic stress in recombinant yeast cells. (**A**,**C**,**F**) Growth curves of yeast transformed by the empty vector pYES2, pYES2-*TtEM1*, and pYES2-*TtEM4* under standard conditions. (**B**,**D**,**E**,**G**,**H**) Growth curves of yeast transformed by the empty vector pYES2, pYES2-*TtEM1*, and pYES2-*TtEM4* after treatment to the heat (42 °C), oxidative stress (H_2_O_2_ 2 mM), cold (−20 °C), osmotic stress (mannitol 1 M), and drought stress PEG (10%), respectively. Growth is measured at a wavelength of 600 nm. Each data point represents an average of three replicates, and error bars represent standard deviations. Statistical significance of the difference was determined by two-way ANOVA: **** *p* < 0.0001, *** *p* < 0.001, and * *p* < 0.05.

The stress-responsiveness of the *TtEM1* and *TtEM4* in wheat and its in vitro protective role led us to examine the potential of these proteins to enhance stress tolerance in yeast. In this regard, the *TtEM1* and *TtEM4* genes were cloned into the pYES2 vector and transformed into BY4741 yeast cells. Growth curves of yeast transformed with pYES2-*TtEM1*, pYES2-*TtEM4*, and the empty vector under control and stress conditions were measured. Under optimal conditions, there was no significant difference in growth between the yeast cells transformed with the empty vector and the yeast cells transformed with pYES2-*TtEM1* or pYES2-*TtEM4.* The OD_600_ values of the transformed strains were nearly identical throughout the time course. For example, at 24 h, OD_600_ values were 2.06 ± 0.008 for the empty vector, 2.05 ± 0.018 for *TtEM1*, and 2.07 ± 0.018 for *TtEM4* (Figure 6A,C,F). Nevertheless, under stress conditions, cells expressing *TtEM1* and *TtEM4* exhibited significantly enhanced tolerance compared to the empty vector. When exposed to oxidative stress (H_2_O_2_), *TtEM1*- and *TtEM4*-expressing yeast exhibited enhanced growth (OD_600_ values at 52 h were 1.45 ± 0.005 and 2.24 ± 0.007, respectively) compared to the cells expressing empty vector (0.31 ± 0.002; Figure 6D). During low-temperature stress (−20 °C), at 32 h, cell viability was significantly higher for yeast cells transformed with *TtEM1* or *TtEM4* (56% and 60%, respectively; Figure 6E). Under mannitol treatment, the transformed yeast cells with pYES2-*TEM1* or pYES2-*TtEM4* exhibited a 18% and 28% higher OD_600_ at 4 h than the control (Figure 6G). Under PEG-induced water deficit, at 4 h, OD_600_ values for *TtEM1*- or *TtEM4*-overexpressed stains were 0.2 ± 0.006 and 0.18 ± 0.007, respectively, whereas control cells reached only 0.15± 0.005 (Figure 6H). Notably, under high-temperature stress, only yeast cells expressing pYES2-*TtEM1* (OD_600_ at 54 h was 2.1 ± 0.06) were able to grow, while yeast cells expressing pYES2-*TtEM4* and the empty vector showed a low growth (OD_600_ values at 54 h were 0.2 ± 0.006 and 0.19 ± 0.003, respectively) (Figure 6B). These results suggest that overexpression of *TtEM1* and *TtEM4* differentially improves the tolerance of transgenic yeast to abiotic stress, indicating that the two genes may function through distinct mechanisms in wheat.

## 3. Discussion

Group 1 *LEA* genes, also known as *Em* genes, have been identified in a wide range of organisms, including plants, bacteria, and animals, highlighting the functional importance of these genes [21]. In this study, we performed the first genome-wide characterization of the *EM* gene family (group 1 LEA) in durum wheat, identifying a relatively high number of eight members compared to those identified in two well-studied model plants, rice and *Arabidopsis*. Analysis of 60 fully sequenced genomes revealed a total of 153 *EM* genes. Currently, a number of *EM* genes have been found in many species. For instance, two *EM* genes were identified in maize (*Zea mays*), in rice, in *Arabidopsis thaliana*, and in *Medicago truncatula*, while six were detected in barley [22]. The number of *EM* genes in wheat was relatively high compared to these species. These findings suggest that differences in the number of *EM* genes might be associated with plants and their ploidy levels [23].

*TtEM* genes were unevenly distributed across the four chromosomes, suggesting a differential gene duplication and loss events, implicated in the expansion of *EM* genes, a major mechanism for gene family expansion in polyploid species like durum wheat [24]. Analysis of their motifs revealed that members of each LEA phylogenetic group contained specific conserved motifs shared with their homologues in other species, such as *A. thaliana*, *M. truncatula*, *H. vulgare*, and *O. sativa*, suggesting that EM proteins probably have specific functions. These results are consistent with previous studies in peanut [25] and brassica species [26], where proteins within the same group exhibited similar motif composition.

Usually, stress responsive-genes have a low intron content [27]; corroborating this hypothesis, all the eight *TtEM* genes contain one intron. This structural feature seems to be a common characteristic among several stress-related gene families. For instance, the trehalose-6-phosphate synthase gene family, which plays an important role in stress response, also exhibited low numbers of introns [28]. Similarly, *ASR* genes in wheat [29] and barley typically contain one intron [30]. Our findings suggest that the compact gene structure of *TtEM*s genes could be an adaptative feature that facilitates rapid transcriptional activation during stress, as explained by the authors of [31], who state that introns can retard regulatory responses by increasing the length of the nascent transcript, leading to an additional energetic burden due to the increase in transcript length.

According to the analysis of physiochemical proprieties, it was found that all the *TtEM* genes encode small proteins with molecular weight ranging between 9.9 and 14.6 kDa, which was consistent with previous finding in other species such as strawberry (4–10 kDa) [32] and [33] tea. In addition, *TtEM* proteins are notably enriched with polar and charged amino acids and depleted of hydrophobic amino acids, a typical feature of intrinsically disordered proteins [34]. Consistent with this, prediction of disordered features of *TtEM* proteins using the PONDOR website tool revealed that the average proportion of disordered structures was higher than 98% for all *TtEM* proteins. These characteristics enable LEA proteins to stabilize and prevent aggregation of the proteins, thereby contributing to various biological roles in response to different environmental conditions [35]. In this current study, *TtEM1* and *TtEM4* proteins were found to be heat-stable proteins and sensitive to protease-meditated proteolysis, revealing their disordered nature. Additionally, both proteins were able to protect LDH activity under different stress conditions, supporting their functional role in stress tolerance. For several LEA proteins, it has been demonstrated that they are unstructured in hydrate solution, but they adopt an α-helix structure during stress [36]. For instance, COR15A, a LEA protein from *Arabidopsis thaliana*, folds into an α-helix in the presence of high concentrations of glycerol [37]. Here, the addition of glycerol results in decreased susceptibility to proteolysis for *TtEM1* and *TtEM4* proteins, suggesting that the two proteins may adopt a predominantly α-helical structure, as indicated by in silico analysis (41.59% and 45.13 of predicted α-helical content for *TtEM1* and *TtEM4*, respectively), likely contributing to their stability under proteolytic treatment.

Previously, many in vitro and in vivo studies have been conducted to investigate the function of the LEA gene under abiotic stress [38]. For example, *LsEm1* from lettuce (*Lactuca sativa*) [18] and *EhEm1* from salt cress (*Eutrema halophilum*) [18] have been shown to protect the LDH enzyme from desiccation treatment. Additionally, overexpression of *RhDHN4* from barley, *TsLEA1* from *Thellungiella salsuginea*, and *ZmLEA5C* from maize has been demonstrated to improve abiotic stress tolerance, such as salt, drought, and oxidative stress, when expressed in yeast systems [39,40,41]. Here, we demonstrated that recombinants expressing *TtEM1* or *TtEM4* proteins grew better under heat, cold, oxidative, and drought stress than yeast not expressing *TtEM1* or *TtEM4* proteins, thus suggesting a functional redundancy between these genes in conferring general stress tolerance. However, a differential stress-specific response has been observed, especially the exclusive high-temperature tolerance conferred by *TtEM1*, suggesting functional divergence between the two genes under high-temperature stress. Such differences enable each gene to contribute to specific aspects of the plant’s stress response. These findings highlight a complementary or specialized role for *TtEM1* or *TtEM4* genes in stress adaptation.

LEA proteins have been predicted to localize in various subcellular compartments and are associated with a range of functions related to stress response, desiccation tolerance, and development [42]. Interestingly, *TtEM* proteins were predicted to localize exclusively in the nucleus, suggesting their role in maintaining the integrity of nuclear structures during stress.

The majority of LEA proteins are upregulated in response to various abiotic stresses [19]. This regulation is largely driven by specific cis-regulatory elements in their promoters. In several plant species, including Sorghum (*Sorghum bicolor*) [43], tomato [44], rye [45], and poplar [46], *LEA* gene promoters have been reported to contain numerous stress-responsive elements, as well as cis-regulatory elements related to plant growth and development. Consistent with these findings, our analysis of the promoter regions of *TtEM* genes revealed the presence of a lot of stress-responsive and growth and development cis-element in durum wheat. This reinforces the notion that *TtEM* gene expression is regulated by stress conditions, as shown in previous studies that demonstrate that the expression of *LEA* genes is frequently induced at different development stages and tissues of plants and by different abiotic stresses, such as salt, drought, temperature, and hormonal treatment [47]. Moreover, our data expand on previous reports by showing that these elements are not only conserved across species but are also functionally relevant in durum wheat, where *TtEM1* and *TtEM4* genes showed a strong transcriptional responses to dehydration and ABA treatment. Interestingly, expression profiling revealed that *TtEM1* and *TtEM4* genes were predominantly expressed in embryos, with markedly lower expression in the other tissues under standard conditions. This tissue-specific expression pattern, combined with their inducibility by abiotic stress, suggests that *TtEM1* and *TtEM4* play critical roles at the intersection of stress adaptation and developmental regulation. Taken together, our study highlights the distinct expression profiles of *TtEM1* and *TtEM4,* providing valuable insights into their potential divergence within the group1 LEA family.

## 4. Materials and Methods

### 4.1. Identification and Analysis of LEA_5 Family Members in Durum Wheat

Group 1 LEA protein members in Durum Wheat were detected using the Ensembl Plants database (https://plants.ensembl.org/index.html, accessed on 24 April 2025) for *Triticum turgidum* (svevo. v1), based on their Pfam IDs (PF00477). Their orthologous genes in *Arabidopsis thaliana*, *Medicago truncatula*, *Hordeum vulgare*, and *Oryza sativa* Japonica were retrieved using the BioMart, along with their nucleotide sequences, protein sequences, and promoter sequences. The chromosomal distribution of these genes was visualized using the MG2C tool (http://mg2c.iask.in/mg2c_v2.1/, accessed on 24 April 2025). Biochemical parameters were analyzed by the ProtParam tool (http://web.expasy.org/protparam/, accessed on 24 April 2025), while secondary structure predictions were performed with the SOPMA tool https://npsa-prabi.ibcp.fr/cgi-bin/npsa_automat.pl?page=/NPSA/npsa_sopma.html (accessed on 26 April 2025). Intrinsic disorder predictions, as well as charge and hydropathy analysis, were conducted using PONDR (http://www.pondr.com, accessed on 24 April 2025). The subcellular localization of the *TtEM* proteins was predicted using the WOLFPSORT tool (https://wolfpsort.hgc.jp/, accessed on 27 April 2025).

### 4.2. Analysis of Phylogeny, Genes Structures, and Conserved Motifs

A multiple-sequence alignment of full-length amino acid sequences of EM proteins from *Triticum turgidum* (*TtEM*), *Arabidopsis thaliana* (*AtEM*), *Medicago truncatula* (*Mt*EM), *Hordeum vulgare* (*HvEM*), and *Oryza sativa* (*OsEM*) was performed using the ClustalW algorithm. The genes were renamed according to their chromosomal locations. A phylogenetic tree was constructed using the neighbor-joining method with 1000 bootstrap replicates in MEGA11 software. Gene structures were visualized using the Gene Structure Display Server (GSDS; http://gsds.cbi.pku.edu.cn/, accessed on 28 April 2025) based on coding sequences (CDSs) and corresponding genomic DNA sequences. Conserved motifs were identified by the Multiple EM for Motif Elicitation (MEME) (https://meme-suite.org/meme/tools/meme, accessed on 29 April 2025).

### 4.3. Cis-Acting Elements and Transcription Factors Analysis

The 2kb upstream sequences of the *TtEM* genes were analyzed using the New PLACE database (https://www.dna.affrc.go.jp/PLACE/?action=newplace, accessed on 29 April 2025) to identify cis-acting regulatory elements. Additionally, the Plant Promoter Analysis Navigator (PlantPAN 4.0; http://plantpan.itps.ncku.edu.tw/plantpan4/index.html, accessed on 30 April 2025) was employed to predict transcription factor families. The results were visualized using TBtools-II v.323 software.

### 4.4. Plant Material, Growth Conditions, and Stress Treatments

The Tunisian durum wheat cultivar *Triticum turgidum* ssp. durum, cv. Om Rabiaa, obtained from the National Institute of Agricultural Research of Tunis (Tunisia), was used to analyze the expression of *TtEM1* and *TtEM4* genes. Seeds were surface-sterilized with 0.5% sodium chloride (NaOCl) for 15 min, rinsed three times with sterile distilled water, and placed on Petri dishes containing a single sheet of Whatman paper for germination. After germination, seedlings were transferred to hydroponic boxes containing nutrient solution and grown under controlled conditions (25 ± 2 °C, 16 h light/8 h dark photoperiod). Twelve-day-old seedlings were subjected to various abiotic stress treatments, 100 μM abscisic acid (ABA), cold stress (4 °C), heat stress (42 °C), 200 mM NaCl (salt stress), osmotic stress (100 mM mannitol), and drought stress (20% PEG 6000). Roots and leaves were collected at two time points (4 h and 24 h post-treatment) for RNA extraction. Additionally, the expression profiles of the *TtEM1* and *TtEM4* genes were analyzed in different tissues after anthesis: ear (Sp1, after 2 days; Sp2, after 4 days; and Sp3, after 7 days), seeds (Sd1, after 4 days; Sd2, after 7 days; and Sd3, after 14 days), endosperm (Ed1, after 14 days), embryo (Em1, after 14 days; and Em2, after 21 days), leaves (OL), stems (St), and anthers (Ant).

### 4.5. RNA Isolation, cDNA Synthesis, and qRT-PCR Analysis

Total RNA was extracted from various tissues of wheat seedlings (cv. Om Rabiaa) using TRIzol reagent (Invitrogen, Carlsbad, CA, USA), according to the manufacturer’s protocol. The extracted RNA was treated with DNase (Thermo Fisher Scientific, Waltham, MA, USA) at 37 °C for 30 min. M-MLV reverse transcriptase (Thermo Fisher Scientific) was used for complementary DNA (cDNA) synthesis. qRT-PCR analysis was conducted to assess *TtEM1* and *TtEM4* expression in Om Rabiaa wheat variety subjected to various stress conditions. qRT-PCR reactions were performed using Bio-Star qPCR-Master mix SYBR blue (GeneON, GroB-Rohrheim, Hesse, Germany) on a Bio-Rad CFX96 real-time PCR detection system. The thermal cycling conditions were as follows: an initial denaturation for 3 min at 95 °C, followed by 40 cycles of 30 sec at 95 °C, and 1 min at 72 °C. Each sample type was analyzed in triplicate and normalized using the cell division control protein (AAA-superfamily of ATPases) (CDC, Ta54227), which has previously been reported as the most stable reference gene for normalizing gene expression in developmental stages and different tissues of wheat [48]. The expression level was determined by applying the 2^−ΔΔCT^ method described by [49].

### 4.6. Expression and Purification of TtEM1 and TtEM4 Proteins

The *TtEM1* and *TtEM4* genes were inserted into the pET28a+ vector, resulting in fusion constructs with an N-terminal his-tag. These genes are placed under the control of an IPTG-inducible T7 promoter to allow for the production of recombinant his-tagged proteins. The resulting constructs, pET28a (+)-*Tt*EM1, and pET28a (+)-*Tt*EM4, were transformed into *E. coli* BL21 cells. Cultures were grown at 37 °C until reaching an OD of 0.8, followed by induction with 1 mM IPTG overnight at 28 °C. Recombinant proteins were purified using HisPur™ Ni-NTA resin (ThermoFisher, Inc., USA), according to recommended protocols. Protein patterns were then analyzed by SDS-PAGE, using Bio Basic Prestained protein ladders as molecular-weight markers. Immunoblot analysis of purified His-*TtEM1*/*TtEM4* proteins was performed using an anti-6x-His Tag monoclonal antibody (Invitrogen), according to the method described by [50]. Protein concentrations were determined using the Bradford assay [51].

### 4.7. Evaluation of Heat Stability and Protease Sensitivity of TtEM1 and TtEM4 Proteins

In order to determine their heat stability, the purified proteins *TtEM1*, *TtEM4* (0.5 mg. mL^−1^), and BSA (0.2 mg. mL^−1^, as a control) were incubated for 30 min and 1 h at 100 °C, centrifuged for 5 min at 10,000 rpm, and the supernatants were then analyzed by SDS-PAGE. For protease sensitivity analysis, equal protein concentrations for *TtEM1*, *TtEM4*, and lysozyme (used as control due to its stability in the presence of protease) were subjected to thermolysin treatment. Aliquots were collected after 30 s, 3 h, and 24 h in the presence of 25% glycerol. Reactions were stopped by the addition of SDS-PAGE loading buffer, followed by incubation at 100 °C for 5 min before electrophoresis on a 15% SDS-PAGE gel.

### 4.8. Lactate Dehydrogenase (LDH) Protection Assays

The Lactate Dehydrogenase Bovine heart (Sigma-Aldrich Inc., St. Louis, MO, USA) was diluted to a final concentration of 20 µg/mL in sodium phosphate buffer (10 mM, pH = 7). The purified *TtEM1* and *TtEM4* or BSA were also diluted to a final concentration of 20 µg/mL in the same buffer. The mixture of LDH and *TtEM* proteins in a molar ratio of 1:20 was totally dehydrated in the speed vac and then rehydrated in the same initial volume. For thermic treatment, the samples were subjected to 45 °C for 30 min or two freeze–thaw cycles (2 min in liquid nitrogen, followed by 5 min at room temperature). After each treatment, the mixture of enzymes and proteins was added to a reaction buffer containing 25 mM Tris-HCl pH 7.5, 2 mM pyruvate (Biomaghreb, Ariana, Tunisia), and 0.15 mM NADH for a final volume of 1 mL. An initial absorbance was measured at 340 nm, and then another measurement was made after 2 min. Each trial was repeated three times, with three technical replicates each, and statistically significant differences were analyzed with GraphPad Prism 9 ANOVA Two-way test with *p* value < 0.0001.

### 4.9. Evaluation of Stress Tolerance in Yeast Cells Expressing TtEM1 or TtEM4

*TtEM1*-pYES2, *TtEM4*-pYES2, and pYES2 were transformed into the yeast host BY4741 strain (Mat a *his3Δ1 leu2Δ met15Δ ura3Δ*) using the lithium acetate method [52]. Growth of the transformed yeast cells was monitored in standard YNB(Gal)-URA medium at 30 °C, under shaking, for the control. For oxidative stress treatment, the medium was supplemented with 2 mM hydrogen peroxide. Osmotic and drought stress were induced by the addition of 1 M mannitol and 10% PEG, respectively. To assess thermal stress tolerance, yeast cells were incubated at 42 °C and −20 °C for 24 h, and then they were transferred to YNB(Gal)-URA medium and incubated at 30 °C. Growth was evaluated with a spectrophotometer by measuring OD_600_.

### 4.10. Statistical Analysis

All experiments and analyses were performed in triplicate. GraphPad Prism version 9.0.0 (121) was used for the graphical representation and analysis of the data using two-way ANNOVA test *p* < 0.05.

## 5. Conclusions

The current study provides a comprehensive analysis of the *EM* gene family in durum wheat. Eight genes were identified and classified into four phylogenetic groups, sharing conserved motifs with their homologues in rice, barley, *Arabidopsis thaliana*, and *Medicago truncatula.* They were unevenly distributed on chromosomes, and their promoter regions harbored a high number of cis-acting elements related to biotic and abiotic response and plant development processes. Among them, *TtEM1* and *TtEM4* were highly expressed in embryos during development in the Om Rabiaa durum wheat variety, and they showed differential expression in leaves and roots under stress conditions. Functional assays demonstrated their ability to preserve LDH activity and improve the growth of transformed yeast during the stress. These findings highlight the role of the *EM* gene family in durum wheat and putative functions of *TtEM* genes in stress and developmental regulation, paving the way for further functional characterization and genetic improvement in wheat and related species.

## Figures and Tables

**Table 1:** Physiochemical proprieties of *Tt*EM proteins.

Name	Number of Amino Acids	Molecular Weight (kDa)	Theoretical pI	Grand Average of Hydropathicity (GRAVY)	Predicted α-Helical Content (%) (SOPMA)	Tendency of Disorder (%) (PONDR)	Predicted Subcellular Localization
TtEM1	113	12.333	5.23	−1.388	41.59	99.23	Nucleus
TtEM2	93	10.017	5.57	−1.403	33.33	98.92	Nucleus
TtEM3	94	10.002	5.29	−1.328	37.23	98.94	Nucleus
TtEM4	133	14.604	5.56	−1.474	45.13	99.25	Nucleus
TtEM5	93	9.962	5.56	−1.375	36.56	98.92	Nucleus
TtEM6	93	9.948	5.25	−1.363	34.41	98.92	Nucleus
TtEM7	114	12.575	5.27	−1.361	42.98	100	Nucleus
TtEM8	124	13.694	5.17	−1.308	45.16	100	Nucleus

**Table 2:** Conserved motifs detected by MEME tools in *TtEM* proteins.

Motif	Logo
1	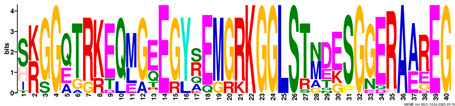
2	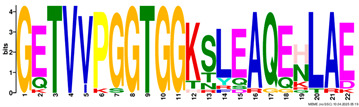
3	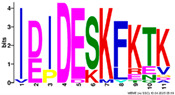
4	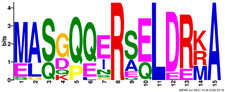
5	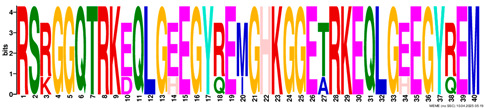
6	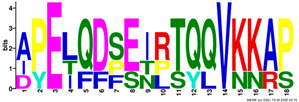
7	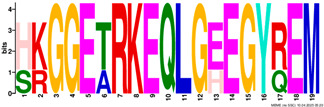
8	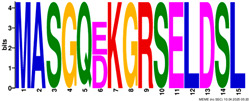
9	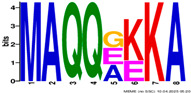
10	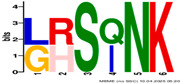

## Data Availability

The data are contained within the article and the Supplementary Materials.

## Supplementary Materials

The following supporting information can be downloaded at https://www.mdpi.com/article/10.3390/plants14182817/s1, Figure S1. Amino acid sequence alignments of *TtEM* proteins. The motif outlined in blue represents the N-terminal region of the sequences, the C-terminal region is highlighted in green, and the orange border indicates the specific motif characteristic of group 1 LEA proteins. Table S1. Chromosomal locations of *EM* genes. Table S2. Cis-regulatory elements on *TtEM* promoters.
