# Supplementary material for: Group 1 LEA Proteins in Durum Wheat: Evolution, Expression, and Roles in Abiotic Stress Tolerance
Source: Plants (Basel). 2025 Sep 9;14(18):2817. doi: 10.3390/plants14182817 (PMC12473993; doi:10.3390/plants14182817)
Supplement: Supplementary file 1 [file plants-14-02817-s001.zip › plants-3747510-supplementary.pdf]

Supplementary Materials

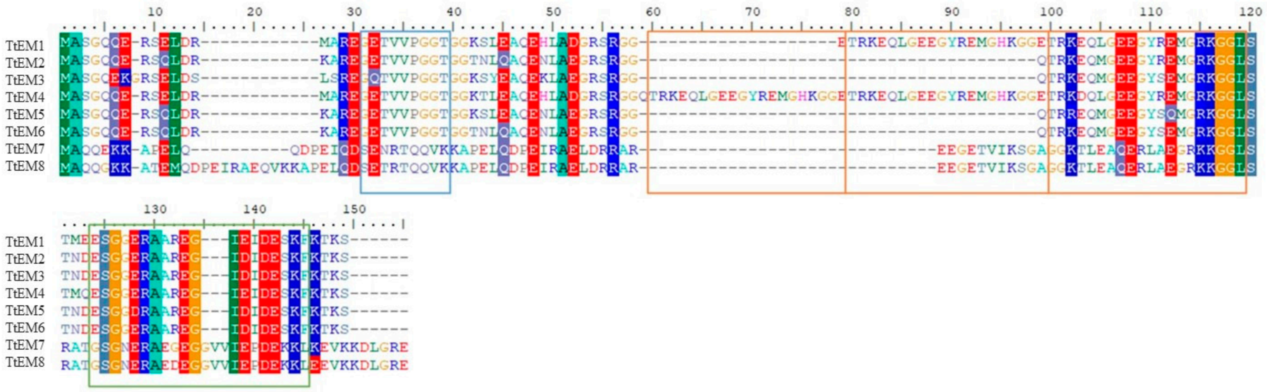

Figure S1. Amino acid sequence alignments of TtEM proteins. The motif outlined in blue represents the N-terminal region of the sequences, the C-terminal region is highlighted in green and the orange border indicates the specific motif characteristic of group 1 LEA proteins.

Table S1. Chromosomal locations of *EM* genes.

Attributed Name	Gene stable ID	Chromosome	Start	End	Chromosome length (pbs)
TtEM1	TRITD1Av1G143760	1A	387171305	387171732	585266722
TtEM2	TRITD1Av1G143780	1A	387196763	387197117	-
TtEM3	TRITD1Bv1G135900	1B	418162076	418162431	681112512
TtEM4	TRITD1Bv1G135980	1B	418294717	418295346	-
TtEM5	TRITD1Bv1G135990	1B	418298770	418299162	-
TtEM6	TRITD1Bv1G136050	1B	418575791	418576162	-
TtEM7	TRITD3Av1G053530	3A	121492782	121493214	746673839
TtEM8	TRITD3Bv1G062840	3B	173298930	173299405	836514780
HvEM1	HORVU.MOREX.r3.1HG0061770	1H	409243732	409244544	516505932
HvEM2	HORVU.MOREX.r3.1HG0061780	1H	409291233	409291633	-
HvEM3	HORVU.MOREX.r3.1HG0061790	1H	409316711	409317259	-
HvEM4	HORVU.MOREX.r3.1HG0061800	1H	409319574	409320481	-
HvEM5	HORVU.MOREX.r3.1HG0061820	1H	409421885	409422498	-
HvEM6	HORVU.MOREX.r3.3HG0246210	3H	113267694	113268462	621516506
OsEM1	Os01g0159600	Chr 1	3124369	3125331	43270923
OsEM2	Os05g0349800	Chr 5	16518577	16519493	29958434
AtEM1	AT2G40170	Chr 2	16779552	16780368	16779552
AtEM2	AT3G51810	Chr 3	19214722	19215831	23459830
MtEM1	gene20818	CM010651.1	5525321	5526029	64763011
MtEM2	gene40990	CM010654.1	32298478	32298985	56236587

Table S2. Cis-regulatory elements on *TtEM* promoters.

	**Cis-regulatory elements**	***TtEM1***	***TtEM2***	***TtEM3***	***TtEM4***	***TtEM5***	***TtEM6***	***TtEM7***	***TtEM8***
ABA Responsive Elements	ABRE	5	17	13	7	3	6	3	11
	ACGTABRE	0	0	0	1	0	0	0	0
	CaE3OSOSEM	0	1	0	0	0	1	0	0
	EMBP1TAEM	0	1	1	0	0	0	0	0
Gibberellin responsive element	GARE	1	1	3	2	2	1	3	0
	MYBGAHV	1	0	0	0	0	0	0	0
	PYRIMIDINEBOX	0	0	1	0	0	2	2	0
	TATCCACHVAL21	1	0	0	0	0	0	1	0
	WRKY71OS	9	20	11	5	15	11	12	13
Cytokinin	ARR1AT	19	9	9	9	16	17	20	9
	CPBCSPOR	0	0	1	0	1	2	0	0
Auxin resonsive element	ARFAT	0	2	1	0	0	1	5	1
	AUXRETGA2GMGH3	0	0	0	0	1	0	0	0
	CATATGGMSAUR	1	2	0	2	0	0	0	2
	D1GMAUX28	0	0	0	0	1	0	0	0
	NTBBF1ARROLB	0	1	1	0	1	1	0	3
Ethylene	GCCCORE	2	1	1	1	1	0	0	1
	ERELEE4	1	0	0	0	0	4	1	0
	LECPLEACS2	0	0	1	1	0	0	0	0
Salicyclic acid	WBOXATNPR1	4	4	4	0	3	4	3	3
Dehydration responsive elements	ABRE	4	12	12	4	7	2	8	13
	ACGTATERD1	12	22	24	14	13	6	22	26
	CBFHV	0	0	0	0	1	0	2	3
	CIACADIANLELHC	2	0	2	1	0	0	1	1
	DRE	3	3	2	0	0	0	4	3
	GCN4OSGLUB1	0	0	1	0	0	0	0	0
	MYB	7	11	14	9	11	9	10	10
	MYC	6	11	15	12	14	8	6	13
Low temperature	CRTDREHVCBF2	2	2	2	0	2	0	2	2
	LTR	8	2	3	1	2	1	8	4
Wound Elicitor and pathogen responsive	AGMOTIFNTMYB2	0	0	0	1	1	0	0	0
	BOXLCOREDCPAL	0	2	1	1	2	0	3	0
	ELRECOREPCRP1	0	1	0	0	1	0	1	0
	GT1GMSCAM4	4	3	5	1	2	5	4	1
	QARBNEXTA	0	0	1	0	0	1	0	0
	WBOX	2	5	4	0	4	1	7	7
Endosperm	-300ELEMENT	0	1	0	2	1	1	0	0
	AACACOREOSGLUB1	1	1	1	0	0	1	1	0
	CANBNNAPA	2	2	2	3	1	0	0	2
	GCN4OSGLUB1	0	0	1	0	0	0	0	0
Pollen	5659BOXLELAT5659	0	1	0	0	0	1	0	0
	GTGANTG10	6	14	18	12	17	15	19	12
	POLLEN1LELAT52	9	5	10	6	7	16	6	3
	QELEMENTZMZM13	0	0	1	0	0	0	2	0
Divers Tissues	BP5OSWX	0	0	0	0	0	0	1	0
	CAATBOX1	17	16	11	18	16	23	11	9
	TATABOX2	0	0	0	0	1	0	0	0
	CIACADIANLELHC	2	0	2	1	0	0	1	1
	CARGCW8GAT	5	4	7	4	3	4	0	0

Seed	ACGTCBOX	0	2	0	0	0	2	0	2
	NAPINMOTIFBN	1	1	2	0	0	0	1	0
	RYREPEAT	6	4	9	2	9	2	2	3
	SPHCOREZMC1	0	0	0	0	1	0	0	0
Root	RHERPATEXPA7	3	6	7	2	7	1	7	9
	ROOTMOTIFTAPOX1	5	3	15	7	8	17	2	1
	SURECOREATSULTR11	2	3	3	3	2	3	10	3
	WUSATAg	0	0	0	1	0	0	0	0
